# Supplementary material for: VS-Cambium-Developer: A New Predictive Model of Cambium Functioning under the Influence of Environmental Factors
Source: Plants (Basel). 2023 Oct 17;12(20):3594. doi: 10.3390/plants12203594 (PMC10609909; doi:10.3390/plants12203594)
Supplement: Supplementary file 1 [file plants-12-03594-s001.zip › plants-2572198-supplementary.pdf]

plants

Article

# VS-Cambium-Developer: A New Predictive Model of Cambium Functioning under the Influence of Environmental Factors

Daria A. Belousova [1,*], Vladimir V. Shishov [2], Alberto Arzac [3], Margarita I. Popkova [1], Elena A. Babushkina [4], Jian-Guo Huang [5], Bao Yang [6] and Eugene A. Vaganov [3]

[1] Siberian Federal University, research department, 660041 Krasnoyarsk, Russia; popkova.marg@gmail.com (M.I.P.)
[2] Siberian Federal University, Institute of Fundamental Biology and Biotechnology, 660041 Krasnoyarsk, Russia; vlad.shishov@gmail.com (V.V.S.)
[3] Siberian Federal University, Institute of Ecology and Geography, 660041 Krasnoyarsk, Russia; aarzac@gmail.com (A.A.); evaganov@sfu-kras.ru (E.A.V.)
[4] Siberian Federal University, Khakass Technical Institute, 655017 Abakan, Russia; babushkina70@mail.ru (E.A.B.)
[5] College of Life Sciences, Zhejiang University, Hangzhou 310058, China; huangjg@scbg.ac.cn (J.-G.H.)
[6] School of Geography and Ocean Science, Nanjing University, Nanjing 210023, China; yangbao@lzb.ac.cn (B.Y.)
* Correspondence: daryadarya1611@gmail.com

Citation: Belousova, D.A.; Shishov, V.V.; Arzac, A.; Popkova, M.I.; Babushkina, E.A.; Huang, J.-G.; Yang, B.; Vaganov, E.A. VS-Cambium-Developer: A New Predictive Model of Cambium Functioning under the Influence of Environmental Factors. *Plants* **2023**, *12*, 3594. https://doi.org/10.3390/plants12203594

Academic Editor(s): Chengliang Sun and, Yiquan Ye

Received: 6 August 2023
Revised: 1 October 2023
Accepted: 6 October 2023
Published: 17 October 2023

Copyright: © 2023 by the authors. Licensee MDPI, Basel, Switzerland. This article is an open access article distributed under the terms and conditions of the Creative Commons Attribution (CC BY) license (https://creativecommons.org/licenses/by/4.0/).

SUPPLEMENTARY

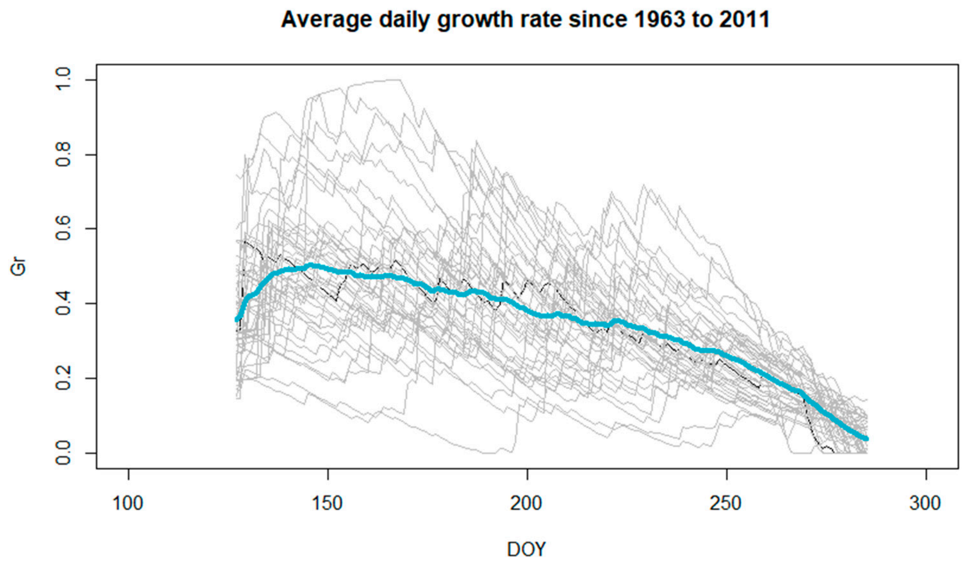

Figure S1. Integral growth rates (gray curves) per year for the period from 1963 to 2011; the average integral cell growth rate calculated by the VS model (blue curve).

We applied the VS-oscilloscope to model the site-averaged response of trees to climate factors. The result is shown in Figure 2S. Calibration period: 1945–1981 (correlation coefficient = 0.56); verification period: 1981–2017 (correlation coefficient = 0.49).

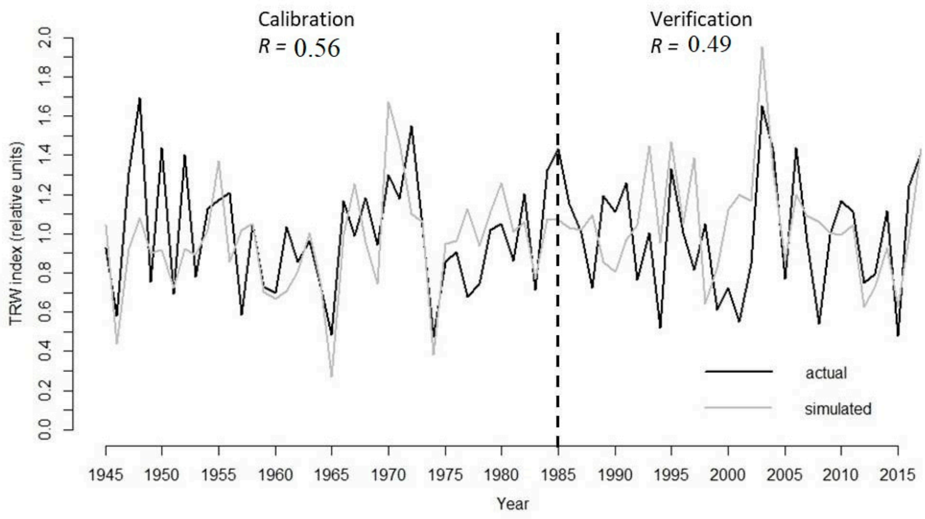

Figure S2. Actual and VS model-simulated growth of the site-averaged response of trees to the influence of climatic factors. Calibration period from 1945 to 1981, verification period from 1982 to 2017.

The average growth sample chosen from a set of samples, for which anatomical measurements were provided, are shown in Figure 3S. In relation to the average growth, we can clearly see fast-growing, slow-growing and medium-growing trees. The medium-growing tree was chosen for the further modeling.

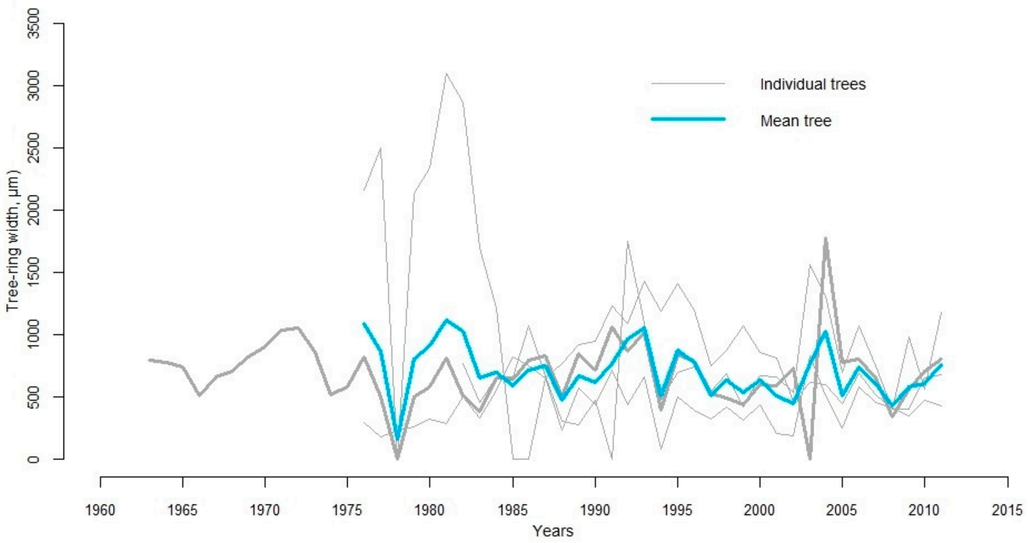

Figure S3. Tree-ring width curves: gray curves represent the five trees (LS1, LS12, LS11, LS13, LS15); the blue curve shows the mean site chronology. The sample selected for further calculations is highlighted by a bold gray curve.

Table S1. Parameters for VS model, which were applied for the selected site.

Parameter	***Value***
Minimum temperature for tree growth	2,50
Lower end of range of optimal temperatures	12,00
Upper end of range of optimal temperatures	21,00
Maximum temperature for tree growth	30,00
Maximum soil moisture for tree growth	1,00
Coefficient of temperature modulation (T)	0,00
Sum of temperature for beginning of soil melting	100,00
First coefficient of soil melting	10,00
Second coefficient of soil melting	0,01
Initial soil moisture	0,20
Maximum daily precipitation for saturated (s)	19,00
Root depth	300
First coefficient for calculation of transpiration	0.13
Second coefficient for calculation of transpiration	0.05
Fraction of precipitation penetrating soil	0.016
Minimum soil moisture for tree growth	0.1
Lower end of range of optimal soil moisture	0.2
Upper end of range of optimal soil moisture	0.55
Growth critical soil moisture	0.8
Coefficient of water drainage from soil	84
Sum of temperature to start growth	84
Initial snowpack	0
Rate of snow melting	53.6
Minimum temperature snow melting	-2
